# Postpartum sacral stress fracture associated with mechanical sacroiliac joint disease: A case report

**DOI:** 10.1097/MD.0000000000011735

**Published:** 2018-08-10

**Authors:** Badii Hmida, Soumaya Boudokhane, Houda Migaou, Amine Kalai, Anis Jellad, Zohra Ben Salah

**Affiliations:** a Department of Radiology, Faculty of Medicine, University Hospital, University of Monastir; b Department of Physical Medicine and Rehabilitation, Faculty of Medicine, University Hospital, University of Monastir, Tunisia.

**Keywords:** osteoarthritis, postpartum, sacroiliac joint, sacrum, stress fracture

## Abstract

**Rationale:**

Stress fractures of the sacrum and mechanical sacroiliac joint disease can occur not only during pregnancy but also postpartum. Mechanical sacroiliac joint disease is common in patients with low back pain but often misdiagnosed by practitioners. The association of the 2 conditions has not been studied yet.

**Patient concerns:**

A 37-year-old woman physiatrist presented with 8-week history of persistent low back and left buttock pain that started in the third trimester of her pregnancy.

**Diagnoses:**

Laboratory investigation, dual-energy x-ray absorptiometry, magnetic resonance imaging, and CT of the pelvic region were performed. The patient was diagnosed with postpartum sacral stress fracture associated with mechanical sacroiliac joint disease.

**Interventions:**

Treatment consisted in pain killers and tailored to a nonweight-bearing period of 3 months.

**Outcomes:**

Painful symptoms disappeared and the patient was able to walk and perform other daily activities normally.

**Lessons:**

Clinician should be aware of the clinical context and the possible association of these 2 conditions in order to undertake an early and appropriate treatment.

## 1 Introduction

Low-back and buttock pain is a common complaint during pregnancy and the postpartum period. This pain is usually attributed to the fetal development and the uterine expansion and spontaneously resolves after delivery. Although rare, stress fractures of the sacrum and sscro-iliitis can occur during this period. They are often misdiagnosed by practitioners and taken for sciatica in late stages of pregnancy. Several case reports of postpartum stress fracture have been reported,^[1–8]^ but rarely associated to mechanical sacroiliac joint disease.

## 2 Case report

A 37-year-old woman physiatrist presented with 8-week history of persistent low back and left buttock pain with difficulty in walking and sitting. Symptoms had started at 34 weeks of pregnancy. She reported similar spontaneously resolving complaints during her previous pregnancy which were attributed to sciatica. Both pregnancies were delivered with cesarean section. Neither history of trauma nor strenuous physical activity was noticed. On examination, the patient was apyretic, 164 cm tall and weighed 48 kg. There were pain and mild tenderness on palpation of the left superior gluteal area. Pain was aggravated during provocative sacroiliac joint maneuvers. Lumbar spine was slightly painful at extension and lateral bending. Neurologic examination was normal. Laboratory investigations were unrevealing with normal values of erythrocyte sedimentation rate, blood calcium and phosphorus levels and kidney and thyroid tests.

**Figure 1:**
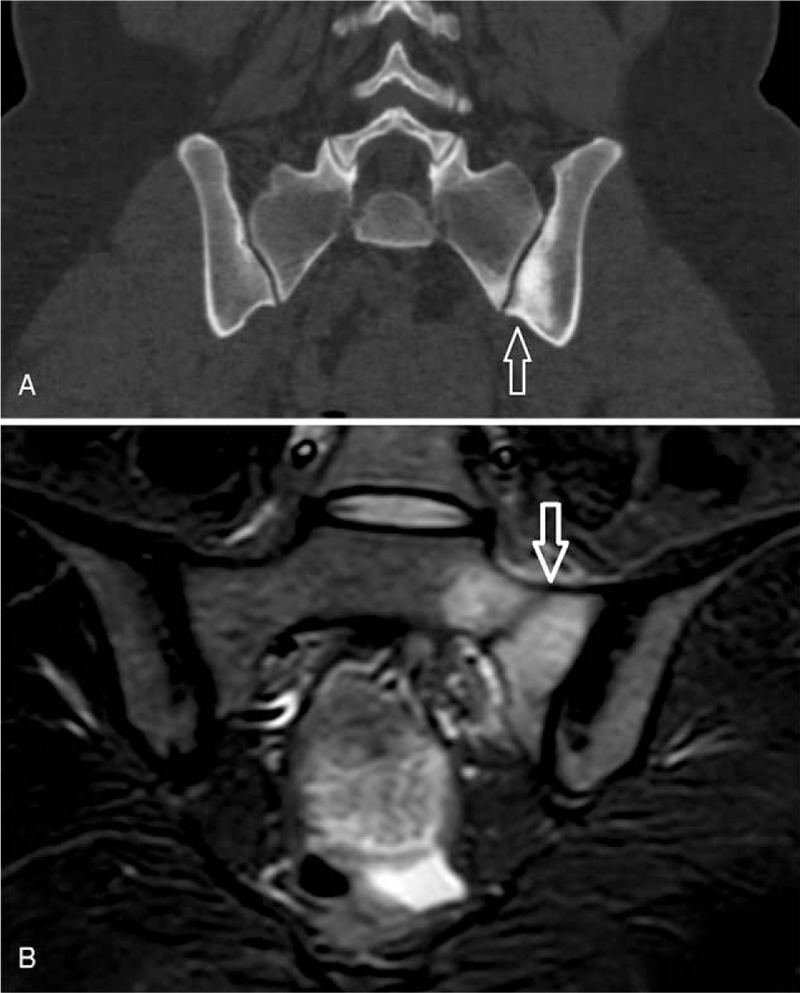
CT scan (A) and T2-STIR MRI (B) images showing respectively a condensation of the left sacroiliac joint (arrow) and a hypointense oblique fracture line surrounded by an area of edema on the left side of the sacrum (arrow). CT = computed tomography, T2-STIR MRI = T2-weighted short-tau inversion recovery magnetic resonance imaging.

Pelvic and lumbar x-rays revealed no bone abnormalities. Pelvic CT scan revealed degenerative changes concordant with a left mechanical sacroiliac joint (SIJ) disease (Fig. 1A). A subsequent MRI showed a hypointense oblique line with marrow edema on the left side of the sacrum consistent with a stress fracture (Fig. 1B). Treatment consisted in relative rest and pain killers and resulted in favorable outcomes. Our case was waived from ethical approval according to our institutional ethical committee. An informed consent was obtained from the patient.

## 3 Discussion

To the best of our knowledge, this is the first report of postpartum sacral stress fracture associated with a mechanical SIJ disease. It was advanced that sacral stress fracture and mechanical SIJ disease are probably underestimated.^[2,9]^ This could be explained by the lack of specificity of symptoms of these conditions and the physician unfamiliarity with sacral stress fractures during pregnancy. Sacroiliitis in the postpartum has been reported to be of infectious or inflammatory origin and in some cases pregnancy was considered as a predisposing factor.^[10]^ In our case there were no infection clinical or radiological signs and the inflammatory origin was very unlikely. Mechanic origin in relation to pregnancy and labor stress was retained. Pregnancy related weight gain, excessive lumbar lordosis, an increase in levels of relaxin hormone, mechanical pelvic stress of delivery during fetal descent especially in cases of rapid vaginal delivery and high birthweight, or pelvic ligaments weakness, lactation related osteopenia and pregnancy osteoporosis, may be incriminated in the development of sacral fractures.^[11,12]^ Analgesics are essential in the treatment of sacral stress fractures as they ensure pain control. They should be used until pain resolves. There is no consensus regarding whether to treat these patients with bed rest or to allow them to ambulate.^[13]^ Early ambulation was suggested by some authors in order to facilitate fracture healing through osteoblast activation and to reduce the risk of immobility related complications.^[13]^ Patients can begin supervised progressive ambulation with assistive devices as long as weight bearing is pain free and tolerated.^[13,14]^

Mechanical SIJ disease is commonly painless. In painful cases, where patients have disability in daily living activities, such as our case, MRI and/or CT scan allow the clinician to rule out sacral fracture. In case of mechanical SIJ disease, MRI findings especially isolated SIJ edema lead commonly to an overdiagnosis of inflammatory sacroiliitis.^[2]^ Mechanical SIJ disease is managed traditionally conservatively with physical therapy, injections and radiofrequency ablation.^[15]^ In our case the association of the 2 conditions led to indicate a relative rest until weight bearing was tolerated with a favorable clinical outcome. The patient recovered normal ambulation within 2 months. In case of persistent low back and/or buttock pain in the postpartum the clinician should prompt investigations to rule out sacral stress fracture even if mechanical SIJ disease is obvious.

## Author contributions

**Conceptualization:** Soumaya Boudokhane.

**Investigation:** Badii Hmida, Houda Migaou, Soumaya Boudokhane, Amine Kalai, Anis Jellad.

**Supervision:** Anis Jellad.

**Writing – original draft:** Badii Hmida, Houda Migaou, Soumaya Boudokhane, Amine Kalai, Anis Jellad, Zohra Ben Salah Frih.
